# Off-the-shelf CAR-T cell therapies for relapsed or refractory B-cell malignancies: latest update from ASH 2023 annual meeting

**DOI:** 10.1186/s13045-024-01550-9

**Published:** 2024-05-06

**Authors:** Yun Kang, Chenggong Li, Heng Mei

**Affiliations:** 1 Institute of Hematology, Union Hospital, Tongji Medical College, Huazhong University of Science and Technology, 1277 Jiefang Avenue, Wuhan, 430022 China; 2 Hubei Clinical Medical Center of Cell Therapy for Neoplastic Disease, Wuhan, 430022 China

## Abstract

Currently, many off-the-shelf chimeric antigen receptor (CAR)-T cell products are under investigation for the treatment of relapsed or refractory (R/R) B-cell neoplasms. Compared with autologous CAR-T cell therapy, off-the-shelf universal CAR-T cell therapies have many potential benefits, such as immediate accessibility for patients, stable quality due to industrialized manufacturing and additional infusions of CAR-T cells with different targets. However, critical challenges, including graft-versus-host disease and CAR-T cell elimination by the host immune system, still require extensive research. The most common technological approaches involve modifying healthy donor T cells via gene editing technology and altering different types of T cells. This article summarizes some of the latest data from preclinical and clinical studies of off-the-shelf CAR-T cell therapies in the treatment of R/R B-cell malignancies from the 2023 ASH Annual Meeting (ASH 2023).

## To the editor

Off-the-shelf chimeric antigen receptor (CAR)-T cells can improve the availability of clinical treatments for patients with relapsed or refractory (R/R) B-cell malignancies by overcoming the limitations of inconsistent quality, high cost, long manufacturing cycles and occasional production failures. The proliferation, persistence and allogeneity of off-the-shelf CAR-T cells, as well as graft rejection by the host immune system, are the primary determinants of the safety and therapeutic effects of these treatments. The two fundamental methods for universal CAR-T cell production are the use of gene editing technology and the adoption of specific T-cell subtypes endowed with universality. We presented several recent studies on the use of off-the-shelf CAR-T cells for the treatment of R/R B-cell malignancies from the 2023 ASH Annual Meeting (ASH 2023).

## Off-the-shelf CAR-T cell therapies based on gene editing

To reduce the risk of graft-versus-host disease (GVHD) and host-versus-graft reactions, knockout (KO) of the genes that encode the T cell receptor alpha (TCR) constant (TRAC) and CD52 by transcription activator-like effector nuclease (TALEN) technology in combination with an anti-CD52 antibody can be used to prevent TCR-mediated recognition of histocompatibility antigens and protect CAR-T cells from lymphodepletion (LD) mediated by an anti-CD52 antibody. [1–3]. Through the application of Cellectis' TALEN® technology, healthy donor T cells are manipulated to generate ALLO-501/ALLO-501A, which is an allogeneic anti-CD19 CAR-T cell line with TRAC and CD52 deletions (Table 1). ALLO-501/ALLO-501A infusions have shown favorable safety and efficacy in patients with R/R large B-cell lymphoma and follicular lymphoma. Twenty-four percent (20/87) of patients experienced cytokine release syndrome (CRS); grade 3–4 CRS and immune effector cell-associated neurotoxicity syndrome (ICANS) both occurred in 1% of patients (Table 2) [1]. There was no significant increase in the occurrence of adverse events associated with enhanced LD. Allogeneic UCART22 and UCART20X22 CAR-T cells were also developed using TALEN technology to disrupt the TRAC gene and CD52 gene. UCART22 Process 2 was administered to three patients with CD22-positive B-cell acute lymphoblastic leukemia (B-ALL), and two patients achieved minimal residual disease (MRD)-negative complete response (CR) without any severe CRS or ICANS [2]. One hundred percent (3/3) of patients with R/R lymphoma who received UCART20 × 22 had a response accompanied by grade 1–2 CRS; neither ICANS nor GVHD occurred. Notably, two patients who previously received anti-CD19 CAR-T-cell therapy achieved metabolic CR [3]. P-BCMA-ALLO1 is a piggyBac® transposon-generated allogeneic CAR-T cell-targeting B-cell maturation antigen (BCMA). The TCRβ 1 chain gene and the β-2 microglobulin gene (B2M) were disrupted by the Cas-CLOVER™ site-specific editing system [4]. The median time from enrollment to infusion was reduced to 7 days. Twenty-one percent (7/22) of patients experienced CRS of low severity, and one had grade 1 ICANS. The overall response rate (ORR) in arms P1 and P2 using optimized LD was 82%, and two patients in arm P2 reached stringent CR [4].

**Table 1:** Properties of off-the-shelf CAR-T cell therapies updated at ASH 2023

	Product	T cell sources	Allogeneic technology	Target antigen	Disease	CAR vector	Current research stage	References
1	ALLO-501/501A	Healthy donor T cells	Cellectis' TALEN® mRNA-mediated TRAC and CD52 KO	CD19	R/R LBCL and FL	Lentiviral transduction	Phase 1 ALPHA (ALLO-501; NCT03939026) and ALPHA2 (ALLO-501A; NCT04416984)	[1]
2	UCART22	Healthy donor T cells	Cellectis' TALEN® mRNA-mediated TRAC and CD52 KO	CD19	R/R CD22+B-ALL	Lentiviral transduction	Phase 1 BALLI-01 (NCT04150497)	[2]
3	UCART20 × 22	Healthy donor T cells	Cellectis' TALEN® mRNA-mediated TRAC and CD52 KO	CD20 and CD22	R/R NHL	Lentiviral transduction	Phase 1/2a NatHaLi-01 (NCT05607420)	[3]
4	P-BCMA-ALLO1	Healthy donor T cells	Cas-CLOVER™ Site-Specific Gene Editing System-mediated TCRβ and B2M KO	BCMA	R/R MM	PiggyBac® DNA Delivery System	Phase 1 P-BCMA-ALLO1-001 (NCT04960579)	[4]
5	RevCAR T	Healthy donor T cells	CRISPR/Cas9-mediated TRAC, HLA-A and MHC II KO	CD19 and CD20	B-cell malignancies	Lentiviral transduction	Preclinical	[5]
6	SC262	Healthy donor T cells	Overexpression of CD47 CRISPR-Cas12b-mediated B2M, CIITA and TRAC KO	CD22	LBCL	Lentiviral transduction	Preclinical	[6]
7	ThisCART19A	Healthy donor T cells	Intracellular retention of TCRαβ/CD3 by a KDEL-tagged anti-CD3 scFV	CD19	R/R B-ALL	Lentiviral transduction	Phase 1 (NCT05350787)	[7]
8	19-28z CAR EBV-CTLs	Healthy donor T cells	EBV specific cytotoxic T lymphocytes	CD19	R/R B-cell malignancies	Lentiviral transduction	Phase 1 (NCT01430390)	[8]
9	ATA3431	Healthy donor T cells	EBV specific cytotoxic T lymphocytes	CD19 and CD20	B-cell malignancies	Retroviral transduction	Preclinical	[9]
10	ADI-001	Healthy donor γδT cells	Vδ1 γδ T cells	CD20	R/R B-NHL	Retroviral transduction	Phase 1 (NCT04735471)	[10]
11	tFas/CD19.CAR-Vδ2 T cells	Healthy donor γδT cells	Vγ9Vδ2 T cells	CD19	B-cell malignancies	NA	Preclinical	[11]
12	anti-CD38-CAR iPS-rCTLs	IPSCs	iPS-derived cytotoxic T lymphocytes	CD38	MM	Retroviral transduction	Preclinical	[12]
13	RJMty19	Healthy donor T cells	DN T cells	CD19	R/R B-NHL	Lentiviral transduction	Phase 1 RJMty19-B-NHL001 (NCT05453669)	[13]

*CAR* chimeric antigen receptor, *iPSCs* induced pluripotent stem cells, *TALEN* transcription activator-like effector nuclease, *KO* knock-out, *CRISPR* clustered regularly interspaced short palindromic repeats, *scFv* single-chain variable fragment, *EBV* Epstein–Barr virus, *DN* double negative, *R/R* refractory or relapse, *LBCL* large-B-cell lymphoma, *FL* follicular lymphoma, *B-ALL *B-cell acute lymphoblastic leukemia, *NHL* non-Hodgkin’s lymphoma, *MM* multiple myeloma, *B-NHL* B cell-NHL, *NA* not available

**Table 2:** Outcomes of clinical studies of off-the-shelf CAR-T cell therapies in relapsed or refractory B-cell malignancies

	Product	Clinical trial	Disease	PTS enrolled	Cohorts	LD regimens	Median time to LD	Median time to infusion	ORR/CR	Long-term outcomes	CRS/NT	GvHD	References
1	ALLO-501 and ALLO-501A	NCT03939026; NCT04416984	R/R LBCL and FL	87	NA	3 to 5-day of F (30 mg/m^2^/d), C (300–500 mg/m^2^/d) and A (39, 60, or 90 mg/d)	3 days (FCA90)	NA	FCA90: ORR 67% (8/12), CR 58% (7/12)	NA	Grade 1/2 CRS: 23% (19/87), Grade 3 CRS: 1% (1/87)	No	[1]
2	UCART22	NCT04150497	R/R CD22 + B-ALL	3	P1: manufacture by external CDMO; P2: manufacture by Cellectis Biologics	FC: 4-day of F (30 mg/m^2^/d), 3-day of C (1 g/m^2^/d) FCA: 3-day of F (30 mg/m^2^/d), C (0.5 g/m^2^/d) and A (20 mg/d)	NA	NA	ORR 67% (2/3), MRD-CR 67% (2/3)	NA	Grade 1/2 CRS: 67% (2/3)	No	[2]
3	UCART20 × 22	NCT05607420	R/R B-NHL	3	NA	3-day of F (30 mg/m^2^/d), C (500 mg/m^2^/d), A (60 mg total)	NA	NA	ORR 100% (3/3), CR 67% (2/3), PR 33% (1/3)	NA	Grade 1/2 CRS: 100% (3/3)	No	[3]
4	P-BCMA-ALLO1	NCT04960579	R/R MM	24	arm S (C 300 mg/m^2^ + F 30 mg/m^2^ X 3 days); arm P1 (C 500 mg/m^2^ + F 30 mg/m^2^ X 3 days); arm P2 (C 1000 mg/m^2^ + F 30 mg/m^2^ X 3 days) arm C (multiple P-BCMA-ALLO1 doses following C 300 mg/m^2^ + F 30 mg/m^2^ X 3 days)	3-day of F (30 mg/m^2^/d) and C (300, 500 or 1000 mg/m^2^/d)	2 days	7 days	Arm S: ORR 0 (0/8) Arm P1: ORR 80% (4/5), VGPR 40% (2/5), PR 40% (2/5) Arm P2: ORR 83% (5/6), sCR 33% (2/6), VGPR 50% (3/6)	NA	Grade 1 CRS: 21% (7/22) Grade 1 ICANS: 4% (1/22)	No	[4]
8	ThisCART19A	NCT05350787	R/R B-ALL	10	NA	5-day of F (30 mg/m^2^/d) and C (300 mg/m^2^/d) and etoposide (100 mg/d)	NA	NA	ORR 100% (7/7), MRD-neg CR/CRi 100% (7/7)	NA	Grade 1/2 CRS: 100% (8/8), Grade ≥ 3 CRS: 25% (2/8) ICANS: 37.5% (3/8)	No	[7]
5	19-28z CAR EBV-CTLs	NCT01430390	R/R B-cell malignancies	16	cohort 1: disease recurrence after allo-HCT or auto-HCT cohort 2: consolidative therapy after auto-HCT cohort 3: consolidative therapy after allo-HCT	NA	NA	NA	NA	mFU 53.3 m, OS at 12 months 81%, OS at 24 months 74%	Grade 1 CRS: 6% (1/16)	Skin GvHD of any grade 12% (2/16), Grade 3 skin GvHD 6%(1/16)	[8]
6	ADI-001	NCT04735471	R/R B-NHL	24	NA	sLD:3-day of F (30 mg/m^2^) and C (500 mg/m^2^) eLD:4-day of F (30 mg/m^2^) and 3-day of C (1000 mg/m^2^)	NA	NA	ORR 7/9 (78%), CR 7/9 (78%)	mFU 1.2–8.8 m, 57.1% pts still CR	Grade 1/2 CRS: 22.2%, Grade 1 ICANS: 11.1%	No	[10]
7	RJMty19	NCT05453669	R/R B-NHL	15	NA	sLD:3-day of F (25 mg/m^2^) and C (500 mg/m^2^) rLD:3-day of F (20 mg/m^2^) and C (300 mg/m^2^)	NA	NA	DL ≥ 3, DCR 100% (5/5), ORR 40% (2/5)	NA	Grade 2 CRS: 9% (1/11)	No	[13]

*R/R* refractory or relapsed, *LBCL* large-B-cell lymphoma, *FL* follicular lymphoma, *B-ALL* B-cell acute lymphoblastic leukemia, *NHL* non-Hodgkin’s lymphoma, *MM* multiple myeloma, *B-NHL* B cell-NHL, *PTS* patients, *NA* not available, *HCT* hematopoietic stem cell transplantation, *LD* lymphodepletion, *F* fludarabine, *C* cyclophosphamide, *A* anti-CD52 antibody, *sLD* standard LD, *eLD* enhanced LD, *rLD* reduced LD, *ORR* overall response rate, *CR* complete response, *MRD* minimal residual disease, *PR* partial response, *VGPR* very good partial response, *sCR* stringent complete response, *CRi* complete response with incomplete marrow recovery, *DL* dose level, *DCR* disease control rate, *mFU* median follow-up, *OS* overall survival, *CRS* cytokine release syndrome, *NT* neurotoxicity, *ICANS* immune effector cell-associated neurotoxicity syndrome, *GVHD* graft-versus-host disease

The reverse universal CAR platform (RevCAR), which targets CD19 and CD20, includes a universal CAR-T cell with KO of the genes encoding the TCR, human leukocyte antigen (HLA)-A and class II major histocompatibility complex transactivator gene (CIITA) and a soluble adaptor called the targeting module (R-TM19/20) [5]. This approaches induces better tumor clearance in B-ALL and lymphoma xenograft models even after CD19-negative relapse [5]. CRISPR-Cas12b is an RNA-guided nuclease platform for genome editing that contains a single RuvC nuclease domain without an HNH domain. It has been used to disrupt B2M, CIIA and TRAC to produce CD22-targeting hypoimmune CAR-T cells (SC262) due to its high efficiency and decreased off-target toxicity in human genome editing [6]. CD47-overexpresing SC262 cells were generated, and these cells can evade killing by NK cells and macrophages by triggering the CD47-SIRPα "don’t eat me" signaling pathway and ultimately elicit robust tumor control in vitro and in vivo [6].

In addition, a novel non-gene-editing strategy to generate ThisCART19A (TCRαβ/CD3 and/or HLA-I intracellular sequestered) was used to transduce a construct encoding a CD19 CAR and a KDEL-tagged anti-CD3 single-chain variable fragment (scFv) into T cells. The secretion of the TCRαβ/CD3 complex from the endoplasmic reticulum was prevented, resulting in the loss of TCR expression. A total of 25% (2/8) of patients with R/R B-ALL experienced grade 3–4 CRS, and 37.5% (3/8) of patients had ICANS, while encouraging efficacy profiles revealed that 100% (7/7) of patients had MRD-negative CR/CR with an incomplete hematologic recovery rate, and 57% (4/7) of patients remained in response with a median follow-up of more than 4.9 months [7].

## Off-the-shelf CAR-T cell therapies originating from specific T-cell types

Epstein‒Barr virus-specific T cells (EBVSTs) can effectively eliminate EB virus-related tumor cells and are considered to be a potential off-the-shelf source due to their restricted TCR repertoire [8, 9]. The 19-28z CAR EBV-CTLs were EBVSTs that were modified with an anti-CD19 CD28-containing CAR and were administered to patients who relapsed or required consolidation therapy after allogeneic or hematopoietic stem cell transplantation [8]. Six percent (1/16) of these patients developed grade 1 CRS, and 18% (3/16) had skin GVHD, including 1 patient with grade 3 GVHD. The 3-year overall survival rate was 74% following multiple infusions. These T cells had lower expression of exhaustion markers, which may contribute to the promising long-term outcomes [8]. ATA3431, which carries a novel CD3ζ signaling domain, 1XX, to extend effector function, is a bispecific CD19- and CD20-targeting allogeneic CAR-T cell based on the EBVST platform that can reliably inhibit B-cell tumor growth according to preclinical evaluations [9].

CAR + γδT cells recognize and kill tumor cells via CAR-dependent and CAR-independent mechanisms, reducing tumor escape without the potential for GVHD. The CD20-targeted universal CAR + Vδ1 γδT cell line ADI-001 exhibited robust dose-dependent expansion and persistence in a phase I trial. Moreover, clinical responses were related to ADI-001 cellular kinetics, regardless of HLA mismatching [10]. Leong et al. altered Vγ9Vδ2 T cells with a reverse fate receptor so that they could avoid activation-induced cell death and augment cytokine signaling via the extracellular domain of Fas and linked intracellular MyD88 [11]. The armed Fas88/CD19.CAR-Vδ2 T cells mediated tumor elimination via massive amplification and persistence in NALM6-bearing mice, suggesting their potential for clinical translation [11]. Induced pluripotent stem (iPS) cells can self-renew, and iPS-derived cytotoxic T lymphocytes (iPS-rCTLs) are expected to survive long term and sustain their cytotoxicity in vivo. IPS-rCTLs transduced with an αCD38 CAR could suppress tumor growth and carry no risk of CD38-mediated fratricide [12]. Double-negative T cells (DNTs) are CD3-positive memory-like T cells that do not have HLA restriction owing to the absence of CD4 and CD8 expression. The first-in-class humanized anti-CD19 allogeneic DNT RJMty19 was well tolerated in 12 patients with non-Hodgkin's lymphoma, and grade 2 CRS was observed in 1 patient without any GVHD or ICANS. The ORR and disease control rate were 40% and 100%, respectively, at the highest dose [13].

## Conclusion and further perspective

Overall, preclinical and clinical research on the use of universal, off-the-shelf CAR-T cells to treat R/R B-cell malignancies has been progressing rapidly, and these cells have demonstrated reliable safety profiles. Most allogeneic CAR-T cell therapies are barely superior to the approved autologous CAR-T cell therapies in terms of efficacy. Gene-editing strategies can transform allogeneic T cells into universal T cell sources through the disruption of critical genes related to allogeneity, such as TRAC, CIITA, and B2M. However, the impact of TCR disruption on the capacity of CAR-T cells to proliferate and kill tumors is still inconclusive. In addition, manufactured CAR-T cells derived from EBVSTs, iPS-rCTLs, γδT cells and DNTs usually equip off-the-shelf CAR-T cells with some inherent properties, including minimal alloreactivity and tumor cytotoxicity. With the increased accessibility and potency of CAR-T cell therapy, establishing platforms for clinical high-yield off-the-shelf CAR-T cell therapy is expected to provide patients with more standardized, dependable, and effective therapeutic approaches.

## Data Availability

Not applicable.
